# Phenotypic Analysis of P‐Wave Morphology as a Key Determinant of Late Recurrence Post‐Ablation in Paroxysmal Atrial Fibrillation

**DOI:** 10.1002/joa3.70285

**Published:** 2026-02-10

**Authors:** Masamichi Yano, Yasuyuki Egami, Noriyuki Kobayashi, Ayako Sugino, Masaru Abe, Mizuki Ohsuga, Hiroaki Nohara, Shodai Kawanami, Kohei Ukita, Akito Kawamura, Koji Yasumoto, Naotaka Okamoto, Yasuharu Matsunaga‐Lee, Masami Nishino

**Affiliations:** ^1^ Division of Cardiology Osaka Rosai Hospital Osaka Japan; ^2^ Department of Cardiology Rinku General Medical Center Osaka Japan; ^3^ Department of Rhythmology University Heart Center Lübeck, University Hospital Schleswig‐Holstein Lübeck Germany; ^4^ Global Center for Medical Engineering and Informatics Osaka University Osaka Japan

**Keywords:** catheter ablation, hierarchical cluster analysis, paroxysmal atrial fibrillation, P‐wave, recurrence

## Abstract

**Background:**

It remains unclear how P‐wave morphology characteristics can be used to stratify the risk of late recurrence after catheter ablation (CA) for atrial fibrillation (AF).

**Methods:**

Patients with paroxysmal AF who underwent an initial CA were enrolled. We investigated the association between P‐wave morphology (P‐wave duration (Pd), PQ interval, P‐wave amplitude (PWA) in leads II, V2, and V6) and late arrhythmia recurrence. Patients were classified into groups using statistical methods, and differences in recurrence and predictive scores for low voltage areas (LVA) among the groups were evaluated.

**Results:**

A total of 1005 paroxysmal AF patients undergoing initial CA were included. Cox regression identified female sex, Pd > 124 ms, PQ > 196 ms, and low PWA in leads II, V2, and V6 as predictors of late recurrence. Hierarchical clustering defined three phenotypes: Phenotype 1 (isolated low PWA), Phenotype 2 (isolated prolonged Pd) and Phenotype 3 (low PWA with prolonged Pd). At 1‐year, cumulative recurrence rates were 10.1% (95% CI 0.8–15.7), 7.0% (4.7–9.6), and 36.2% (30.8–42.3) for Phenotypes 1–3; at 3‐year, rates were 17.4% (12.8–23.3), 10.2% (7.4–14.0), and 61.2% (54.8–67.6). Phenotype 3 showed the highest risk, with HRs of 4.84 (95% CI 3.42–6.84) versus Phenotype 1 and 7.44 (4.34–12.8) versus Phenotype 2 (both *p* < 0.001). Phenotype 3 also had higher DR‐FLASH and APPLE scores than the other phenotypes.

**Conclusions:**

Low PWA across multiple leads (II, V2, and V6), especially when combined with prolonged Pd, correlates with late arrhythmia recurrence and suggests the potential presence of LVA.

## Graphical abstract

Phenotype 3, defined by the combination of prolonged Pd and low PWA in leads II, V2, and V6—representing orthogonal planes—was associated with the highest risk of late arrhythmia recurrence after CA, compared to Phenotype 1 (isolated low PWA) and Phenotype 2 (isolated prolonged Pd). This phenotype also demonstrated the highest scores in LVA prediction models (DR‐FLASH and APPLE), suggesting advanced atrial remodeling. Clinically, patients with Phenotype 3 were characterized by relatively older age, higher body mass index, hypertension, renal dysfunction, elevated BNP levels, and enlarged left atrial and ventricular diameters. BNP, brain natriuretic peptide; CA, catheter ablation; LVA, low voltage area; Pd, P‐wave duration; PWA, P‐wave amplitude.

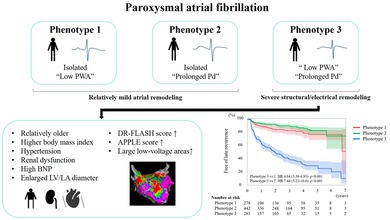

## 1 Introduction

Atrial remodeling is triggered by various stimuli such as hypertension, left ventricular systolic/diastolic dysfunction, and mitral valve disease, and plays a significant role in the development of atrial fibrillation (AF) [1]. Key histological changes in atrial remodeling include the loss of cardiomyocytes and the formation of interstitial fibrosis, which lead to electrical conduction abnormalities in the atria. As a result, these changes are reflected as prolonged P‐wave duration (Pd) and reduced P‐wave amplitude (PWA) on electrocardiograms (ECG) [2]. Previous studies have shown that P‐wave parameters indicating intra‐atrial conduction heterogeneity are associated with arrhythmia recurrence after catheter ablation (CA) for AF [3, 4]. Specifically, the prolongation of Pd in sinus rhythm has been shown to be associated with an increased risk of arrhythmia recurrence after CA in patients with persistent AF [4]. Additionally, a reduction in PWA has also been reported to correlate with arrhythmia recurrence after CA for AF [3]. However, P‐wave assessment using a single lead may not accurately reflect the electrical activity of the atria. To accurately evaluate the heart's electrical activity in a three‐dimensional space, orthogonal leads are necessary. Since the standard 12‐lead ECG does not represent a complete orthogonal coordinate system, it has been suggested that the information from lead II represents the frontal plane, lead V2 provides information from the anterior plane and lead V6 corresponds to the horizontal plane [5]. These leads can be combined to approximate an orthogonal coordinate system and assess the magnitude of the P‐wave vector in three‐dimensional space. On the other hand, the PQ interval represents the combination of intra‐atrial conduction time and the delay in excitation within the atrioventricular node. Abnormalities in the PQ interval are associated not only with pathologies of the atrioventricular node but also with intra‐atrial abnormalities [6], assuming that infranodal conduction is intact. While Pd, PWA, and PQ interval are considered key parameters, it remains unclear how these factors, including P‐wave morphology, are related to arrhythmia recurrence after CA for AF. The aim of this study was to statistically classify patients with paroxysmal AF (PAF) based on the morphology of the Pd, PQ interval, and PWA in each lead during sinus rhythm, and to evaluate the differences in patient characteristics and late arrhythmia recurrence risk after CA between these subgroups.

## 2 Methods

### 2.1 Study Population

PAF patients who underwent an initial CA were enrolled between October 2014 and April 2022 from the ORAF (Osaka Rosai Atrial Fibrillation ablation) registry [7]. Patients who underwent a 12‐lead ECG 1‐day before the procedure and exhibited no P‐wave in all leads (suspected junctional rhythm), ectopic P‐wave (negative P‐wave in leads I, II, and V1), or atrial pacing were excluded from this study. The procedure was in accordance with the “Declaration of Helsinki” and the ethical standards of the responsible committee on human experimentation. This study was approved with an exemption from requiring individual informed consent by the Osaka Rosai Hospital Ethics Committee, given its retrospective observational design. Informed consent for data use was obtained from all patients upon hospital admission.

### 2.2 Electrocardiogram

The ECG was recorded with a paper speed of 25 mm/s and a gain setting of 0.1 mV/mm on a Fukuda Denshi (Tokyo, Japan) machine. Pd, PQ interval, and P‐wave amplitudes in leads II, V2, and V6 were measured automatically using a computerized system (Fukuda Denshi) with 4× magnification. High‐ and low‐pass filters were set at 0.5 and 75 Hz, respectively. The location and morphology of the P wave were visually confirmed by two independent cardiologists, and any discrepancies were resolved by consensus.

### 2.3 Echocardiography and Laboratory Measurements

All patients underwent transthoracic echocardiography before CA using a 5‐MHz multiplane probe, interpreted by experienced physicians blinded to CA outcomes. Examinations followed American Society of Echocardiography guidelines [8]. Transesophageal echocardiography was performed pre‐procedure to exclude left atrium (LA) or LA appendage thrombi. Blood samples were taken 1 day before CA.

### 2.4 Catheter Ablation

Medication management, sedation, and anticoagulation were performed as previously described in our report [7]. Radiofrequency CA was performed with a 3.5‐mm irrigated contact force‐sensing catheter and CARTO3 mapping (Biosense‐Webster, Irvine, CA, USA); procedures before July 2018 were guided by contact force, and those thereafter by ablation index. Following PVI, non‐PV triggers were induced with isoproterenol and/or adenosine triphosphate and ablated if reproducible, with additional lesions applied at the operator's discretion. Cryoballoon ablation was performed using a fourth‐generation 28‐mm balloon with a mapping catheter (Medtronic, Minneapolis, MN, USA), with freezing generally maintained for 180 s if isolation occurred within 60 s, and extended up to 300 s if not. Laser balloon ablation (HeartLight, CardioFocus Inc., Marlborough, MA, USA) was conducted with a compliant balloon system guided by endoscopic visualization, delivering circumferential energy (5.5–12 W/cm, 20–30 s per lesion), with balloon repositioning to ensure complete PV isolation. Non‐PV trigger induction was not performed in cryoballoon or laser balloon cases. Low‐voltage areas (LVAs) were defined as sites with a bipolar peak‐to‐peak voltage of < 0.5 mV. Mapping points were automatically acquired using the criteria of cycle length stability, catheter position stability, and point density. The LVA size was manually measured on each voltage map. We confirmed the detection of non‐PV foci (firing to AF), and we attempted to locate the spontaneous onset of the ectopic beats initiating AF in the baseline state or after an infusion of isoproterenol (up to 30 μg/min).

### 2.5 Follow‐Up and Clinical Outcomes

Patients underwent continuous ECG monitoring for 3 days post‐CA and 12‐lead ECGs at each visit. A 24‐h Holter ECG was performed at 6 and 12 months, then annually. Most patients visited private clinics every 2–4 weeks and were encouraged to monitor pulse/rhythm via smartphone/tablet and report symptoms. Follow‐up included clinical interview, ECG, blood tests, Holter or 1‐week portable ECG, and transthoracic echocardiography. Late arrhythmia recurrence (≥ 3 months post‐CA) was defined as AF/atrial tachycardia documented on ECG or lasting > 30 s on Holter/portable ECG.

We investigated whether P‐wave morphology (Pd, PQ interval, and PWA in leads II, V2, and V6) was associated with late recurrence of arrhythmia. Based on these factors, we classified the enrolled patients into several groups using a statistical method. We then evaluated differences in baseline characteristics, late arrhythmia recurrence after CA, predictive scores for LVAs including DR‐FLASH and APPLE scores [9, 10], and electrophysiological findings in repeat ablation among the groups.

### 2.6 Statistical Analysis

JMP 17 statistical software (SAS Institute Inc., Cary, NC, USA) was used for the statistical analysis. Continuous variables were expressed as the median [interquartile range]. A normality test was performed for continuous variables by a Shapiro–Wilk *W*‐test. Categorical data were expressed as the number (percentage) and were compared using the chi‐squared test. The Kruskal–Wallis test was used for intergroup differences of continuous variables. The Bonferroni method was used to adjust *p*‐values in multiple testing. To evaluate the incremental prognostic value of P‐wave phenotype classification beyond conventional clinical variables and individual P‐wave parameters, we performed a stepwise Cox proportional hazards regression analysis in three stages: Step 1: a base model was constructed including only established clinical predictors associated with recurrence risk. Step 2 (a–e): each of the five P‐wave parameters (P‐wave duration, PQ interval, and P‐wave amplitude in leads II, V2, and V6) was added individually to the clinical model to assess its independent association with recurrence. Step 3: a final model was constructed including the clinical predictors and the P‐wave phenotype classification. As the phenotype classification was derived from the five P‐wave parameters, we did not include the individual P‐wave variables in the same model in Step 3 to avoid multicollinearity. Model fit was assessed using the Akaike Information Criterion (AIC), with lower values indicating better fit. The prognostic predictability of late arrhythmia recurrence, those of Pd, PQ interval and PWA in leads II, V2 and V6 were evaluated with receiver operating characteristic (ROC) curve analysis. Multivariable logistic regression was used to test whether the P‐wave phenotype classification independently predicts high DR‐FLASH or APPLE scores, including all other score components except LA diameter, which was excluded due to multicollinearity. Hierarchical clustering was conducted using data which agglomerated and classified clusters using Ward's method based on the associated factors of late arrhythmia recurrence. The optimal number of clusters was determined using the cubic clustering criterion (CCC) in JMP, selecting the cluster number with the highest CCC value. Kaplan–Meier curves show either Log‐rank *p*‐values or Cox regression results, with 95% confidence intervals (CIs) for recurrence probabilities. A value of *p* < 0.05 was considered to be statistically significant in the present study.

## 3 Results

### 3.1 Patient Characteristics

This study included a total of 1005 patients with PAF from the ORAF registry who underwent an initial CA. The baseline characteristics of the enrolled patients are summarized in Table 1. The median age was 71 years, and the proportion of female patients in the overall population was 41.2%. The proportion of females was significantly higher in patients with late arrhythmia recurrence compared to those without. ECG findings indicated that the Pd and the PQ interval were significantly longer and the PWA in leads II, V2, and V6 were lower in patients with late arrhythmia recurrence compared to those without. Additionally, LA diameter was significantly larger in patients with late arrhythmia recurrence than in those without.

**TABLE 1:** Baseline patient characteristics and procedural characteristics.

	Overall population (*n* = 1005)	Recurrence (−) (*n* = 754)	Recurrence (+) (*n* = 251)	*p*
Clinical data				
Age, years	71 [64, 77]	71 [64, 77]	71 [64, 77]	0.645
Female	414 (41.2)	288 (38.2)	126 (50.2)	< 0.001
BMI, kg/m^2^	23.5 [21.2, 26.0]	23.5 [21.1, 25.7]	23.7 [21.6, 26.4]	0.128
Hypertension	593 (59.0)	445 (59.0)	148 (59.0)	0.988
Diabetes	158 (15.7)	122 (16.2)	36 (14.3)	0.488
Chronic heart failure	91 (9.1)	60 (8.0)	31 (12.3)	0.036
Stroke	93 (9.3)	67 (8.9)	26 (10.4)	0.486
Dyslipidemia	330 (32.8)	247 (32.8)	83 (33.1)	0.928
CHA2DS2 VASc score				
0	82 (8.2)	62 (8.2)	20 (8.0)	0.203
1	178 (17.7)	135 (17.9)	43 (17.1)	
≥ 2	745 (74.1)	557 (73.9)	188 (74.9)	
Laboratory data				
BUN, mg/dL	16 [13, 19]	16 [13, 19]	17 [14, 19]	0.226
Creatinine, mg/dL	0.8 [0.7, 1.0]	0.8 [0.7, 1.0]	0.8 [0.6, 1.0]	0.451
Hemoglobin, g/L	13.5 [12.3, 14.5]	13.6 [12.3, 14.5]	13.2 [12.1, 14.3]	0.018
BNP, pg/mL	54.9 [24.1, 134.0]	50.4 [23.1, 109.1]	77.8 [33.7, 193.9]	< 0.001
Electrocardiographic parameters				
P‐wave duration, ms	116 [108, 128]	115 [104, 124]	132 [124, 140]	< 0.001
PQ interval, ms	176 [160, 196]	176 [160, 192]	184 [168, 208]	< 0.001
PWA (II), mV	0.15 [0.12, 0.18]	0.15 [0.12, 0.19]	0.13 [0.10, 0.15]	< 0.001
PWA (V2), mV	0.10 [0.08, 0.13]	0.11 [0.09, 0.13]	0.09 [0.07, 0.11]	< 0.001
PWA (V6), mV	0.09 [0.08, 0.11]	0.10 [0.09, 0.11]	0.08 [0.06, 0.09]	< 0.001
Echocardiographic parameters				
LVDd, mm	47 [45, 51]	47 [45, 50]	48 [45, 51]	0.395
LVDs, mm	29 [26, 31]	29 [26, 31]	29 [26, 32]	0.627
LVEF, %	70 [65, 73]	70 [65, 73]	70 [65, 73]	0.920
LA diameter, mm	42 [38, 46]	41 [38, 45]	43 [40, 48]	< 0.001
Medication				
AAD	193 (19.2)	144 (19.1)	49 (19.5)	0.883
ACEI/ARB	357 (35.5)	271 (35.9)	86 (34.3)	0.630
Beta‐blocker	389 (38.7)	270 (35.8)	119 (47.4)	0.001
Statin	306 (30.5)	223 (29.6)	83 (33.1)	0.298
Ablation procedure				
RFCA/Cryoballoon/Laser balloon	615 (61.2)/312 (31.0)/78 (7.8)	459 (60.9)/236 (31.3)/59 (7.9)	156 (62.2)/76 (30.3)/19 (7.6)	0.754
Procedure time	145 [100, 193]	141 [99, 185]	158 [106, 207]	0.004
LAPWI	43 (4.3)	30 (4.0)	13 (5.2)	0.416
SVCI	57 (5.7)	44 (5.8)	13 (5.2)	0.697
Atrial tachycardia ablation	26 (2.6)	18 (2.4)	8 (3.2)	0.489

*Note:* Continuous data are presented as the median (interquartile range). Categorical variables are presented as numbers (percentage).

Abbreviations: AAD, anti‐arrhythmic drug; ACEI, angiotensin converting enzyme inhibitor; AF, atrial fibrillation; ARB, angiotensin II receptor blocker; BMI, body mass index; BNP, brain natriuretic peptide; BUN, blood urea nitrogen; LA, left atrium; LAPWI, left atrial posterior wall isolation; LVDd, left ventricular end‐diastolic diameter; LVDs, left ventricular end‐systolic diameter; LVEF, left ventricular ejection fraction; PWA, P‐wave amplitude; RFCA, radiofrequency catheter ablation; SVCI, superior vena cava isolation.

### 3.2 P‐Wave Parameters, Hierarchical Clustering and Phenogroups

**FIGURE 1:**
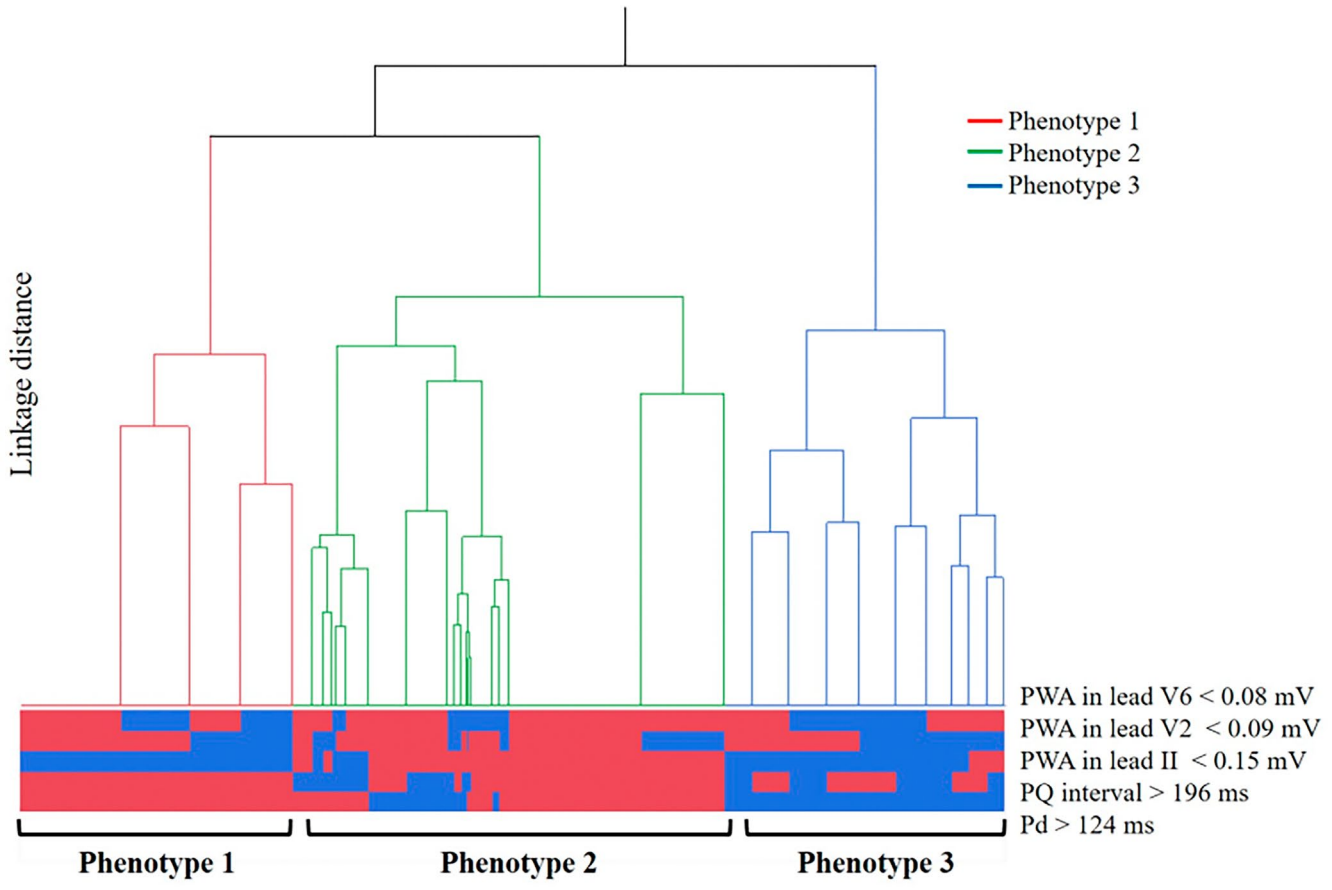
Hierarchical clustering analysis. Dendrogram of hierarchical clustering using three factors in PAF patients after CA. Pd, P‐wave duration; PWA, P‐wave amplitude.

**FIGURE 2:**
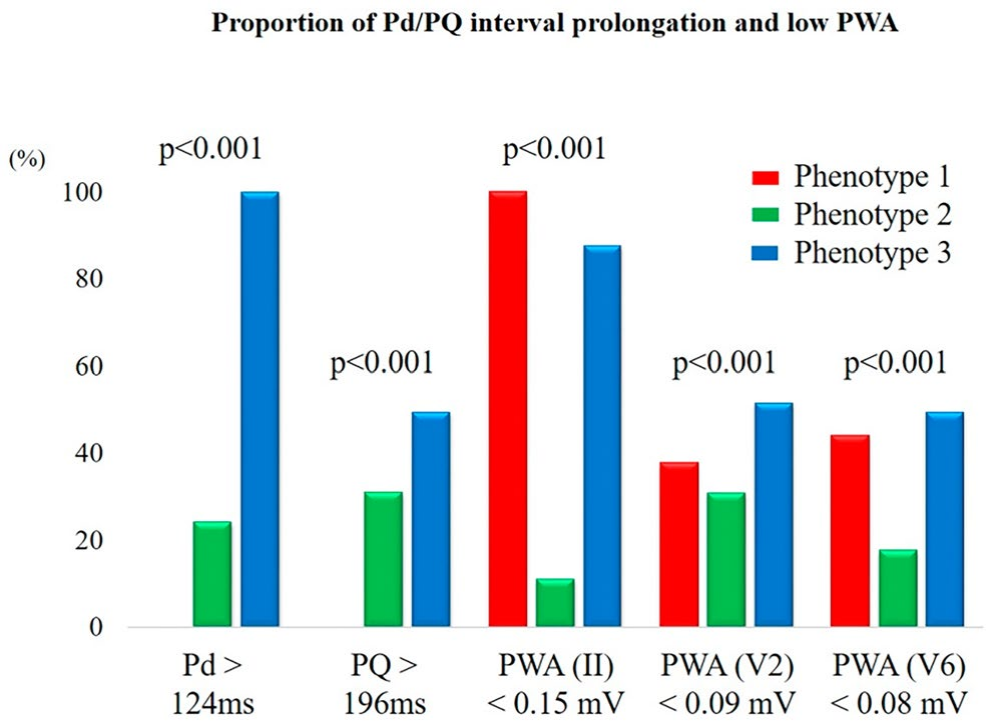
P‐wave abnormal morphology. Proportion of Pd/PQ interval prolongation and low PWA among the phenogroups. Pd, P‐wave duration; PWA, P‐wave amplitude.

ROC analysis identified optimal cutoffs for predicting recurrence: Pd = 124 ms (AUC = 0.83), PQ = 196 ms (AUC = 0.60), PWA in lead II = 0.15 mV (AUC = 0.69), V2 = 0.09 mV (AUC = 0.63), and V6 = 0.08 mV (AUC = 0.81) (Figure S1). Using Pd > 124 ms, PQ > 196 ms, and low PWA in leads II, V2, and V6, three phenogroups were identified (Figure 1): Phenotype 1: isolated low PWA, Phenotype 2: isolated prolonged Pd, and Phenotype 3: combined low PWA and prolonged Pd. Baseline characteristics are in Table 2. Phenotype 3 included older individuals, with higher BMI, hypertension, creatinine, and BNP. Echocardiography showed larger left ventricular end‐diastolic diameter, left ventricular end‐systolic diameter, and LA diameter in Phenotype 3. ECG findings showed the longest Pd and PQ in Phenotype 3 (vs. Phenotypes 1 and 2, both *p* < 0.01). PWA in leads II and V6 was significantly lower in Phenotypes 1 and 3 vs. Phenotype 2 (both *p* < 0.01). In lead V2, PWA was lowest in Phenotype 3 (vs. both, p < 0.01). Figure 2 shows the proportion of patients exceeding each cutoff by phenotype.

**TABLE 2:** Characteristics of three phenotypes.

	Phenotype 1 (*n* = 278)	Phenotype 2 (*n* = 442)	Phenotype 3 (*n* = 285)
Clinical data			
Age, years	70 [60, 77]	71 [65, 76]	73 [65, 78]**
Female	132 (47.5)	168 (38.0)*	114 (40.0)
BMI, kg/m^2^	23.5 [21.1, 26.6]	23.2 [20.9, 25.3]	24.1 [21.7, 26.7]*, ^a^
Hypertension	159 (57.2)	242 (54.8)	192 (67.4)*, ^a^
Diabetes	49 (17.6)	63 (14.3)	46 (16.1)
Chronic heart failure	30 (10.8)	28 (6.3)	33 (11.6)
Stroke	20 (7.2)	45 (10.2)	28 (9.8)
Dyslipidemia	97 (34.9)	129 (29.2)	104 (36.5)
CHA2DS2 VASc score			
0	24 (8.6)	41 (9.3)	17 (6.0)
1	64 (23.0)	718 (16.1)	43 (15.1)
≥ 2	190 (68.3)	330 (74.7)	225 (78.9)
Laboratory data			
BUN, mg/dL	16 [13, 19]	16 [13, 19]	17 [14, 20]*
Creatinine, mg/dL	0.76 [0.65, 0.94]	0.81 [0.66, 0.94]	0.83 [0.70, 1.01]**, ^a^
Hemoglobin, g/L	13.5 [12.2, 14.4]	13.5 [12.4, 14.5]	13.4 [12.2, 14.5]
BNP, pg/mL	46.5 [22.3, 123.2]	54.9 [24.0, 111.7]	63.7 [29.0, 154.7]**, ^a^
ECG parameters			
P‐wave duration, ms	108 [100, 116]	116 [104, 120]**	132 [124, 140]**, ^a^
PQ interval, ms	168 [152, 176]	178 [160, 200]**	192 [176, 212]**, ^a^
PWA (II), mV	0.12 [0.10, 0.14]	0.18 [0.16, 0.20]**	0.13 [0.10, 0.15]**, ^a^
PWA (V2), mV	0.11 [0.08, 0.13]	0.11 [0.09, 0.13]	0.09 [0.08, 0.11]**, ^a^
PWA (V6), mV	0.09 [0.07, 0.10]	0.10 [0.09, 0.12]**	0.09 [0.07, 0.10]^a^
Echocardiographic parameters			
LVDd, mm	47 [45, 51]	47 [45, 50]	49 [46, 52]**, ^a^
LVDs, mm	28 [26, 31]	28 [26, 31]	29[27, 32]**, ^a^
LVEF, %	70 [65, 73]	70 [65, 73]	70 [65, 73]
LA diameter, mm	42 [39, 46]	41 [37, 44]**	44 [40, 48]**, ^a^
Medication			
AAD	35 (12.6)	89 (20.1)**	69 (24.2)**
ACEI/ARB	98 (35.3)	142 (32.1)	117 (41.1)^a^
Beta‐blocker	104 (37.4)	145 (32.8)	140 (49.1)**, ^a^
Statin	82 (29.5)	128 (29.0)	96 (33.7)
Ablation procedure			
RFCA/Cryoballoon/Laser balloon	166 (59.7)/89 (32.0)/21 (7.6)	264 (59.7)/140 (31.7)/38 (8.6)	185 (64.9)/81 (28.4)/19 (6.7)

*Note:* Continuous data are presented as the median (interquartile range). Categorical variables are presented as numbers (percentage).

Abbreviations: AAD, anti‐arrhythmic drug; ACEI, angiotensin converting enzyme inhibitor; ARB, angiotensin II receptor blocker; BMI, body mass index; BNP, brain natriuretic peptide; BUN, blood urea nitrogen; LA, left atrium; LVDd, left ventricular end‐diastolic diameter; LVDs, left ventricular end‐systolic diameter; LVEF, left ventricular ejection fraction; RFCA, radiofrequency catheter ablation.

* *p* < 0.05 versus Phenotype 1.

** *p* < 0.01 versus Phenotype 1.

^a^ *p* < 0.01 versus Phenotype 2.

### 3.3 Association of Phenotypes With Late Arrhythmia Recurrence After CA

**FIGURE 3:**
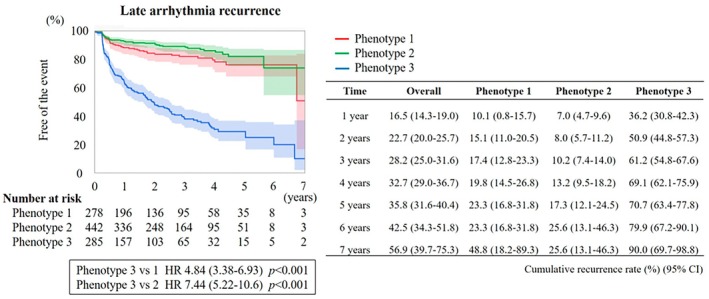
Risk of late arrhythmia recurrence following CA. Kaplan–Meier analysis of late arrhythmia recurrence across the phenogroups. CI, confidence interval; HR, hazard ratio.

The median follow‐up was 722 [288, 1119] days, during which late arrhythmia recurrence occurred in 251 patients (25.0%). Kaplan–Meier analysis demonstrated that Phenotype 3 was associated with a significantly higher risk of late arrhythmia recurrence after CA compared to both Phenotype 1 and Phenotype 2 (Figure 3). At 1‐year, cumulative recurrence rates were 10.1% (95% CI 0.8–15.7), 7.0% (95% CI 4.7–9.6), and 36.2% (95% CI 30.8–42.3) for Phenotypes 1–3; at 3‐year, rates were 17.4% (95% CI 12.8–23.3), 10.2% (95% CI 7.4–14.0), and 61.2% (95% CI 54.8–67.6). Recurrence rates were comparable among radiofrequency CA, cryoballoon, and laser balloon ablation (Figure S2), supporting the independence of our findings from ablation modality. Stepwise Cox proportional hazards regression analysis demonstrated that in Step 1, a model including only clinical predictors was constructed (AIC = 3064.1) (Table 3). In Step 2, each P‐wave parameter—P‐wave duration, PQ interval, and P‐wave amplitude in leads II, V2, and V6—was added individually, all showing significant associations with recurrence risk, with the lowest AIC observed for the model including P‐wave duration (AIC = 2910.3) (Table 3). In Step 3, a model combining clinical predictors with the P‐wave phenotype classification exhibited the lowest AIC (2898.1), and Phenotype 3 was associated with a significantly higher risk of recurrence compared to Phenotypes 1 and 2 (HR 4.84 and 7.44, both *p* < 0.001) (Table 3). These results suggest that phenotype classification provides incremental prognostic value beyond both clinical predictors and individual P‐wave parameters due to the lower AIC values in Step 3.

**TABLE 3:** Stepwise Cox proportional hazard analysis for late arrhythmia recurrence.

Variables	Step 1 HR (95% CI)	Step 2a HR (95% CI)	Step 2b HR (95% CI)	Step 2c HR (95% CI)	Step 2d HR (95% CI)	Step 2e HR (95% CI)	Step 3 HR (95% CI)
	*p* + clinical factors	*p* + Pd	*p* + PQ interval	*p* + PWA in II	*p* + PWA in V2	*p* + PWA in V6	*p* + Phenotype
Age	1.00 (0.98–1.01) *p* = 0.799	0.99 (0.98–1.01) *p* = 0.270	1.00 (0.98–1.01) *p* = 0.580	1.00 (0.98–1.01) *p* = 0.639	1.00 (0.99–1.01) *p* = 0.998	1.00 (0.98–1.01) *p* = 0.903	0.99 (0.98–1.01) *p* = 0.253
Female	1.54 (1.14–2.09) *p* = 0.005	1.86 (1.37–2.52) *p* < 0.001	1.60 (1.18–2.16) *p* = 0.003	1.48 (1.09–2.02) *p* = 0.012	1.58 (1.17–2.14) *p* = 0.003	1.37 (1.01–1.86) *p* = 0.041	1.74 (1.27–2.37) *p* < 0.001
BMI	1.01 (0.96–1.04) *p* = 0.908	0.99 (0.95–1.03) *p* = 0.482	1.00 (0.96–1.04) *p* = 0.862	1.00 (0.96–1.04) *p* = 0.868	1.00 (0.96–1.04) *p* = 0.967	1.01 (0.97–1.05) *p* = 0.692	0.98 (0.94–1.02) *p* = 0.429
Hypertension	0.89 (0.66–1.20) *p* = 0.457	0.85 (0.62–1.16) *p* = 0.295	0.90 (0.67–1.21) *p* = 0.487	0.88 (0.65–1.18) *p* = 0.390	0.85 (0.63–1.15) *p* = 0.288	0.95 (0.70–1.29) *p* = 0.741	0.77 (0.56–1.05) *p* = 0.104
Diabetes mellitus	0.74 (0.51–1.08) *p* = 0.123	0.88 (0.61–1.28) *p* = 0.511	0.77 (0.53–1.12) *p* = 0.167	0.75 (0.51–1.10) *p* = 0.136	0.71 (0.49–1.05) *p* = 0.083	0.75 (0.51–1.09) *p* = 0.133	0.86 (0.59–1.26) *p* = 0.439
Ablation methods							
RFCA (control)	1.00	1.00	1.00	1.00	1.00	1.00	1.00
Cryoballoon	1.02 (0.76–1.36) *p* = 0.910	0.99 (0.74–1.32) *p* = 0.959	1.04 (0.78–1.39) *p* = 0.780	1.01 (0.76–1.35) *p* = 0.941	1.01 (0.76–1.35) *p* = 0.941	0.87 (0.65–1.16) *p* = 0.350	0.99 (0.74–1.32) *p* = 0.942
Laser balloon	1.47 (0.90–2.40) *p* = 0.122	1.73 (1.06–2.83) *p* = 0.027	1.51 (0.92–2.46) *p* = 0.100	1.55 (0.95–2.52) *p* = 0.082	1.52 (0.93–2.48) *p* = 0.095	1.61 (0.98–2.63) *p* = 0.060	1.65 (1.00–2.69) *p* = 0.058
Log BNP	1.27 (0.91–1.76) *p* = 0.160	1.13 (0.80–1.59) *p* = 0.479	1.25 (0.90–1.74) *p* = 0.188	1.39 (0.99–1.93) *p* = 0.053	1.32 (0.94–1.84) *p* = 0.102	1.18 (0.84–1.66) *p* = 0.345	1.24 (0.88–1.75) *p* = 0.211
Creatinine	1.09 (1.00–1.17) *p* = 0.036	1.04 (0.96–1.12) *p* = 0.335	1.07 (0.99–1.16) *p* = 0.084	1.07 (0.98–1.15) *p* = 0.103	1.07 (0.98–1.15) *p* = 0.093	1.07 (0.99–1.16) *p* = 0.078	1.02 (0.93–1.10) *p* = 0.703
Hemoglobin	0.97 (0.88–1.07) *p* = 0.502	0.96 (0.87–1.07) *p* = 0.481	0.97 (0.88–1.07) *p* = 0.513	0.99 (0.90–1.10) *p* = 0.880	1.00 (0.91–1.11) *p* = 0.976	1.00 (0.90–1.11) *p* = 0.985	0.98 (0.88–1.09) *p* = 0.704
LVEF	1.00 (0.99–1.02) *p* = 0.619	1.00 (0.98–1.01) *p* = 0.746	1.00 (0.99–1.02) *p* = 0.727	1.01 (0.99–1.02) *p* = 0.423	1.00 (0.99–1.02) *p* = 0.628	1.01 (0.99–1.02) *p* = 0.233	0.99 (0.98–1.01) *p* = 0.494
LA diameter	1.05 (1.02–1.08) *p* < 0.001	1.03 (1.01–1.06) *p* = 0.013	1.04 (1.02–1.07) *p* = 0.001	1.04 (1.01–1.06) *p* = 0.007	1.05 (1.03–1.08) *p* < 0.001	1.03 (1.01–1.06) *p* = 0.016	1.02 (1.00–1.05) *p* = 0.068
ACEI/ARB	0.93 (0.69–1.25) *p* = 0.621	0.91 (0.67–1.23) *p* = 0.519	0.91 (0.67–1.22) *p* = 0.520	0.88 (0.65–1.19) *p* = 0.415	0.95 (0.70–1.28) *p* = 0.724	0.89 (0.66–1.20) *p* = 0.450	0.89 (0.66–1.21) *p* = 0.468
β‐blocker	1.26 (0.96–1.64) *p* = 0.094	1.05 (0.81–1.37) *p* = 0.723	1.21 (0.92–1.58) *p* = 0.173	1.15 (0.88–1.51) *p* = 0.300	1.31 (1.00–1.72) *p* = 0.047	1.16 (0.88–1.52) *p* = 0.290	1.05 (0.81–1.38) *p* = 0.696
P‐wave duration > 120 ms		6.03 (4.43–8.22) *p* < 0.001					
PQ interval > 196 ms			1.55 (1.18–2.04) *p* = 0.002				
PWA (II) < 0.15 mV				2.81 (2.05–3.86) *p* < 0.001			
PWA (V2) < 0.09 mV					2.40 (1.85–3.10) *p* < 0.001		
PWA (V6) < 0.08 mV						4.60 (3.47–6.08) *p* < 0.001	
Phenotype							
Phenotype 3 vs. 1							4.84 (3.38–6.93) *p* < 0.001
Phenotype 3 vs. 2							7.44 (5.22–10.6) *p* < 0.001
Akaike Information Criterion	3064.1	2910.3	3056.7	3018.6	3022.2	2942.5	2898.1

Abbreviations: ACEI, angiotensin converting enzyme inhibitor; ARB, angiotensin II receptor blocker; BNP, brain natriuretic peptide; CI, confidence interval; HR, hazard ratio; LA, left atrium: LVEF, left ventricular ejection fraction; Pd, P‐wave duration; PWA, P‐wave amplitude; RFCA, radiofrequency catheter ablation.

### 3.4 Relationship Between Phenogroups and LVA Prediction Scores

**FIGURE 4:**
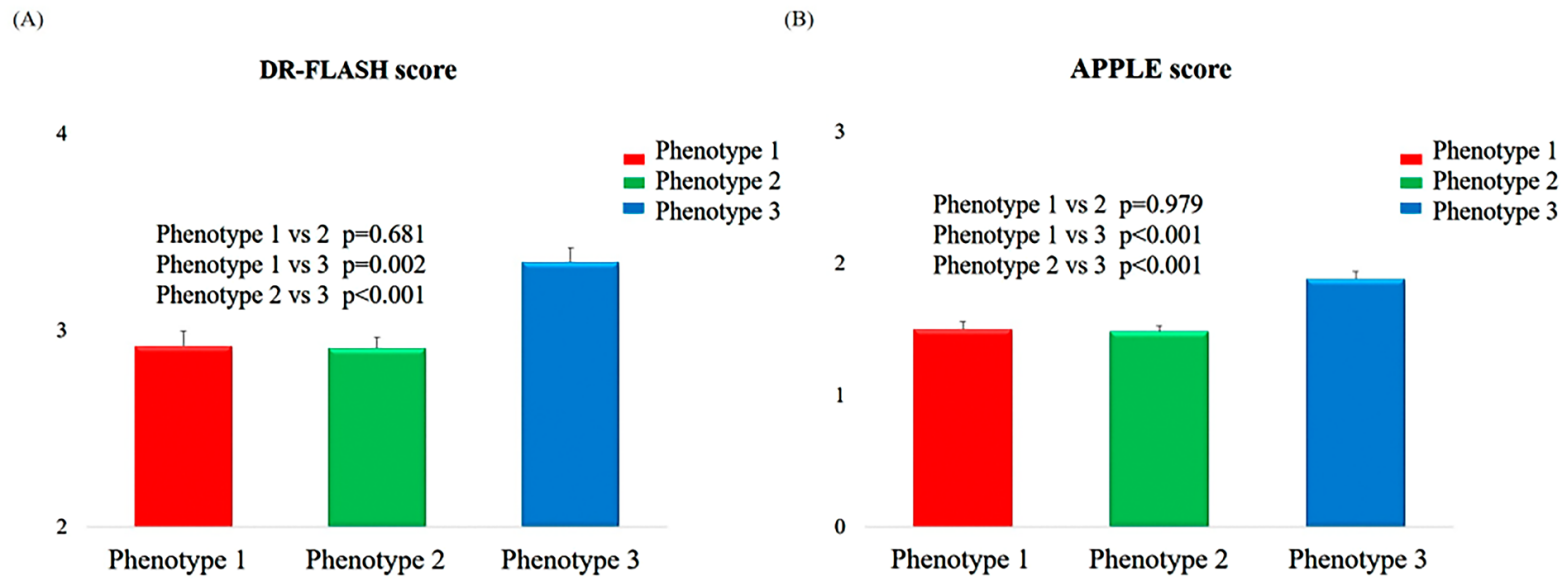
LVA predictive score. DR‐FLASH score across the phenogroups. APPLE score across the phenogroups.

The DR‐FLASH score and APPLE score, estimating the likelihood of underlying LVA in the LA, are shown in Figure 4. Both scores were significantly higher in Phenotype 3 than Phenotype 1 and Phenotype 2 in the DR‐FLASH score (*p* = 0.002 vs. Phenotype 1 and *p* < 0.001 vs. Phenotype 2) and APPLE score (*p* < 0.002 vs. Phenotype 1 and p < 0.001 vs. Phenotype 2).

### 3.5 Electrophysiological Findings in Repeat Ablation

**FIGURE 5:**
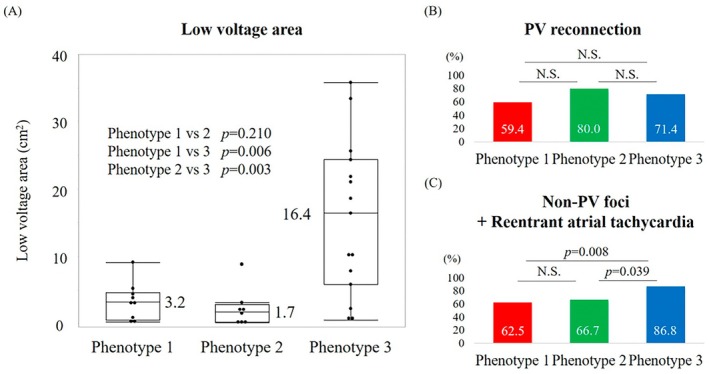
Electrophysiological findings in repeat ablation. LVA across the phenogroups. Proportion of PV reconnection. Proportion of non‐PV foci/reentrant AT. AT, atrial tachycardia; LVA, low‐voltage area; PV, pulmonary vein.

Among 251 patients with recurrence, 153 underwent repeat ablation. Phenotype 3 showed significantly larger LVAs than Phenotypes 1 or 2. Detailed LA voltage mapping was available in 32 patients, again confirming larger LVAs in Phenotype 3 (Figure 5). The phenotype distribution was 32, 30, and 91 patients for Phenotypes 1, 2, and 3, respectively. PV reconnection occurred in 108 patients (19, 24, and 65 by phenotype), while non‐PV foci and/or reentrant atrial tachycardia were detected in 119 (20, 20, and 79). Although PV reconnection did not differ across groups, non‐PV foci/reentrant AT were more frequent in Phenotype 3 (Figure 5). Multivariable logistic regression further showed that phenotype classification independently predicted high DR‐FLASH and APPLE scores beyond their clinical components (Table S1), indicating the additive prognostic value of the ECG‐based classification.

## 4 Discussion

### 4.1 Main Findings

The main findings in the present study were that (1) Pd prolongation and low PWA in leads II, V2, and V6 were significantly and independently associated with an increased risk of late arrhythmia recurrence after CA, (2) Phenotype 3, characterized by both low PWA and prolonged P, had the higher risk of late arrhythmia recurrence after CA compared to Phenotype 1 “Isolated low PWA” and Phenotype 2 “Isolated prolonged Pd” and (3) Phenotype 3 showed the highest scores of LVA prediction models (DR‐FLASH and APPLE scores) among the groups and also had the largest LVAs. These results suggested that low PWA in leads II, V2, and V6, when approximated in the orthogonal coordinate system, is significantly associated with late arrhythmia recurrence after CA and the presence of PWA in multiple leads is more strongly correlated with late arrhythmia recurrence. Furthermore, when accompanied by prolonged Pd, this suggests not only late arrhythmia recurrence but also the presence of LVA.

### 4.2 Alternations in P‐Wave Morphology

The Pd prolongation suggests delayed impulse propagation and heterogeneity of atrial conduction [11]. While PWA and its association with outcomes following cardioversion or CA have been extensively reported [5, 6], the most optimal lead for evaluating intra‐atrial conduction remains unclear. Low PWA in lead I has been associated with LA remodeling and independently predicts arrhythmia recurrence after radiofrequency CA in PAF patients [3]. Conversely, low PWAs in leads II and V1 have been linked to AF recurrence after cardioversion [12]. This discrepancy may be attributed to differences in the electrophysiological and anatomical characteristics of the atrium, including atrial dilatation, fibrosis and impaired conduction pathways. Therefore, P‐wave evaluation in a single lead may not accurately represent the magnitude of atrial electrical activation. We selected lead II as representing the frontal plane, lead V2 as the anterior plane, and lead V6 as the horizontal plane in the standard 12‐lead ECG. The three electrocardiographic leads of these approximate orthogonal coordinate systems are all associated with clinical outcomes (Table 3). These findings suggest that comprehensive evaluation of P‐wave characteristics, including both duration and amplitude across multiple leads, may provide valuable insights for predicting arrhythmia recurrence after CA.

### 4.3 Characteristics of Phenogroups

The low voltage substrate in the LA is associated with conduction delay within the LVA and reflects a prolongation of the Pd on the 12‐lead surface ECG [13]. The P wave is thought to represent the overall electrical activity of the atrium, and the presence of LVA may lead to a reduction in atrial muscle mass, resulting in a decrease in the PWA [14, 15]. Additionally, as atrial fibrosis progresses locally, conduction may become dispersed in multiple directions, which could cause a decrease in the PWA in specific leads compared to normal conduction. AF requires both abnormal pacemaker activity, resulting from a decrease in resting membrane potential and enhanced intrinsic automaticity, and triggered activity, represented by ectopic activity induced by intracellular Ca^2+^ overload, as well as reentry caused by local conduction delays and shortened refractory periods in the myocardial tissue [16]. Once AF occurs, structural changes such as myocardial hypertrophy, cell death, atrial enlargement, and fibrosis are observed. Fibrosis, in particular, is the most crucial pathological change for the maintenance of AF [17], forming a substrate for reentry through reduced conduction velocity and spatial inhomogeneity [1, 18]. In Phenotype 3, the predictive score for LVA was significantly higher, suggesting that the combination of Pd prolongation and decreased PWA is associated with the presence of atrial fibrosis, which is thought to promote the initiation and maintenance of AF.

Phenotype 3 was associated with older age, larger BMI, a higher proportion of hypertension, impaired renal function, and reduced cardiac function (elevated BNP, left ventricular enlargement, and LA enlargement) compared to Phenotype 1 and Phenotype 2. Age and renal function have been linked to LVA in studies such as DR‐FLASH and APPLE [9, 10], while hypertension and obesity cause left ventricular diastolic dysfunction [19, 20], leading to increased LA pressure and progression of LA remodeling. These findings indicate the importance of careful monitoring in patients with prolonged Pd and reduced PWA in multiple leads.

Phenotype 1 exhibited LA enlargement, with a higher proportion of females compared to Phenotype 2 (Table 2). Previous studies have reported that female patients with AF have lower peak atrial longitudinal strain on echocardiography compared to males, suggesting a progression of LA fibrosis [21]. In Phenotype 1, this female‐specific characteristic, which may lead to LA enlargement through atrial myocardial stretch, appears to play a significant role in promoting the progression of atrial fibrosis.

Phenotype 2 exhibits a smaller LA diameter compared to phenotype 1, indicating an earlier stage of LA remodeling. Despite Pd prolongation, absence of significant PWA reduction may reflect limited fibrosis. Previous studies have reported that shortening of the atrial effective refractory period, increased spatial heterogeneity, and reduced conduction velocity induced by high‐frequency atrial pacing occur prior to the development of atrial fibrosis [22, 23]. Among these stress‐induced changes, abnormal expression of connexin‐40, which has been correlated with Pd prolongation, represents a key example of impaired intercellular electrical coupling [24]. These alterations create a substrate for functional reentry and contribute to the maintenance of AF.

### 4.4 Clinical Implications

Even brief AF recurrences may signal a progressive increase in AF burden linked to stroke and heart failure [25]. P‐wave phenotype classification can identify patients with non‐PV foci or LVA in LA; it helps us determine ablation strategies, leading to improvement of long‐term outcomes. The combination of prolonged Pd and low PWA in multiple leads serves as a non‐invasive marker for advanced atrial remodeling, a key substrate for PAF. Phenotype 3, characterized by both prolonged Pd and low PWA, is also associated with higher predictive scores for LVA. These findings suggest that these electrocardiographic markers can help identify patients with substantial structural remodeling, increasing their risk for AF recurrence and guiding more targeted post‐ablation care. P‐wave characteristics more directly reflect LA electrical remodeling than other non‐invasive markers and show stronger predictive value for arrhythmia recurrence than several clinical risk factors in our Cox analysis. Moreover, compared with laboratory or echocardiographic examinations, ECG can be obtained easily, repeatedly, and at lower cost, supporting the practicality of incorporating P‐wave characteristics into risk models for recurrence prediction and personalized care. Integrating these electrocardiographic markers with clinical factors such as age, BMI, and renal function enables clinicians to more accurately predict late arrhythmia recurrence and the presence of LVA. This comprehensive risk stratification approach holds promise for tailoring personalized treatment plans, ultimately improving outcomes and enhancing the management of PAF patients in the chronic phase. In particular, the higher prevalence of non‐PV foci in Phenotype 3 suggests that balloon‐based ablation may be less effective in this subgroup and that radiofrequency point‐by‐point ablation may be more suitable. Future studies should explore how these electrocardiographic markers can inform therapeutic decisions, including the need for additional ablation, antiarrhythmic drug therapy, and repeat procedures.

### 4.5 Limitations

This study has several limitations. First, a major limitation of this study is its retrospective, single‐center design. While the sample size is relatively large, the generalizability of the findings may be limited due to potential center‐specific practices and patient characteristics. Therefore, external validation in prospective, multicenter cohorts is warranted to confirm the applicability of our results to broader populations. In particular, external validation of the ECG cutoffs (Pd = 124 ms, PQ = 196 ms, PWA: II = 0.15 mV, V2 = 0.09 mV, V6 = 0.08 mV) and P‐wave phenotypes is needed to confirm their clinical applicability for risk stratification. Second, selection bias cannot be excluded, as only patients who underwent initial CA for PAF were included. Third, although we identified independent factors associated with Pd and PWA in II, V2, and V6, and classified patients into three clusters using hierarchical clustering, unmeasured confounders might have influenced the clustering results.

## 5 Conclusion

In this study, low PWA in leads II, V2, and V6 and prolonged Pd are significant risk factors for late arrhythmia recurrence following CA. Additionally, Phenotype 3, characterized by both low PWA and prolonged Pd, exhibited a higher risk of late arrhythmia recurrence compared to Phenotype 1 (Isolated low PWA) and Phenotype 2 (Isolated prolonged Pd). The presence of low PWA across multiple leads in leads II, V2, and V6, as approximated in the orthogonal coordinate system, especially when combined with prolonged Pd, not only correlates with late arrhythmia recurrence but also indicates the potential presence of LVA.

## Funding

The authors have nothing to report.

## Conflicts of Interest

The authors declare no conflicts of interest.

## Supporting information


**Data S1:** joa370285‐sup‐0001‐DataS1.zip.

## Data Availability

The data sets analyzed in this study will be shared upon reasonable request to the corresponding author after the approval of the Institutional Review Board in Osaka Rosai Hospital.
