# Supplementary material for: Phenotypic Analysis of P‐Wave Morphology as a Key Determinant of Late Recurrence Post‐Ablation in Paroxysmal Atrial Fibrillation
Source: J Arrhythm. 2026 Feb 10;42(1):e70285. doi: 10.1002/joa3.70285 (PMC12891814; doi:10.1002/joa3.70285)
Supplement: Supplementary file 1 — Data S1: joa370285‐sup‐0001‐DataS1.zip. [file JOA3-42-e70285-s001.zip › supinfo/joa370285-sup-0002-FigureS1-S2@Supplementary figure legend.docx]

**Supplementary figure legends.**

**Supplementary figure 1. ROC analysis of P-wave morphology parameters in predicting late arrhythmia recurrence**

(A) ROC analysis of Pd (B) ROC analysis of PQ interval (C) ROC analysis of PWA in lead II (D) ROC analysis of PWA in lead V2 (E) ROC analysis of PWA in lead V6

Pd, P-wave duration; PWA, P-wave amplitude; ROC, receiver operating characteristics

**Supplementary figure 2. Risk of late arrhythmia recurrence following CA**

Kaplan-Meier analysis of late arrhythmia recurrence between radiofrequency CA, cryoballoon ablation and laser balloon ablation

CI, confidence interval.
