# Supplementary material for: Phenotypic Analysis of P‐Wave Morphology as a Key Determinant of Late Recurrence Post‐Ablation in Paroxysmal Atrial Fibrillation
Source: J Arrhythm. 2026 Feb 10;42(1):e70285. doi: 10.1002/joa3.70285 (PMC12891814; doi:10.1002/joa3.70285)
Supplement: Supplementary file 1 — Data S1: joa370285‐sup‐0001‐DataS1.zip. [file JOA3-42-e70285-s001.zip › supinfo/joa370285-sup-0004-TableS1@Supplementary Table 1.docx]

**Supplementary Table 1** Multivariate logistic regression analysis for high DR-FLASH and APPLE scores

|  | DR-FLASH score = 5, 6 | | | APPLE score = 3, 4 | | |
| --- | --- | --- | --- | --- | --- | --- |
| Variables | OR | 95% CI | *p* value | OR | 95% CI | *p* value |
| Phenotype 3 vs Phenotype 1, 2 | 2.34 | 1.35-4.06 | 0.002 | 1.92 | 1.30-2.83 | 0.001 |
| Age | 1.11 | 1.06-1.16 | <0.001 | 1.14 | 1.11-1.17 | <0.001 |
| Female | 129.4 | 34.8-481.4 | <0.001 |  |  |  |
| Hypertension | 71.8 | 13.6-380.0 | <0.001 |  |  |  |
| Diabetes mellitus | 111.3 | 30.6-404.4 | <0.001 |  |  |  |
| Creatinine | 1.24 | 1.03-1.51 | 0.023 | 1.66 | 1.42-1.98 | <0.001 |
| LVEF |  |  |  | 0.92 | 0.90-0.94 | <0.001 |

CI, confidence interval; LVEF, left ventricular ejection fraction; OR, odds ratio
