# Supplementary figures and images for: Phenotypic Analysis of P‐Wave Morphology as a Key Determinant of Late Recurrence Post‐Ablation in Paroxysmal Atrial Fibrillation
Source: J Arrhythm. 2026 Feb 10;42(1):e70285. doi: 10.1002/joa3.70285 (PMC12891814; doi:10.1002/joa3.70285)

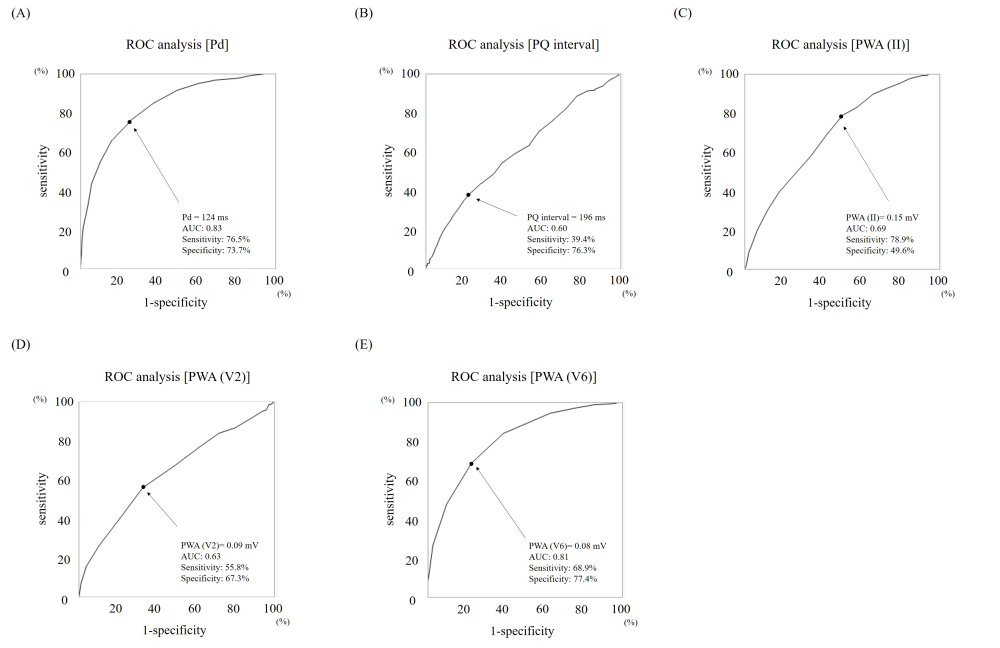

Supplement: Supplementary file 1 — Data S1: joa370285‐sup‐0001‐DataS1.zip. [file JOA3-42-e70285-s001.zip › supinfo/joa370285-sup-0001-FigureS1@Supplementary Figure 1.tif]

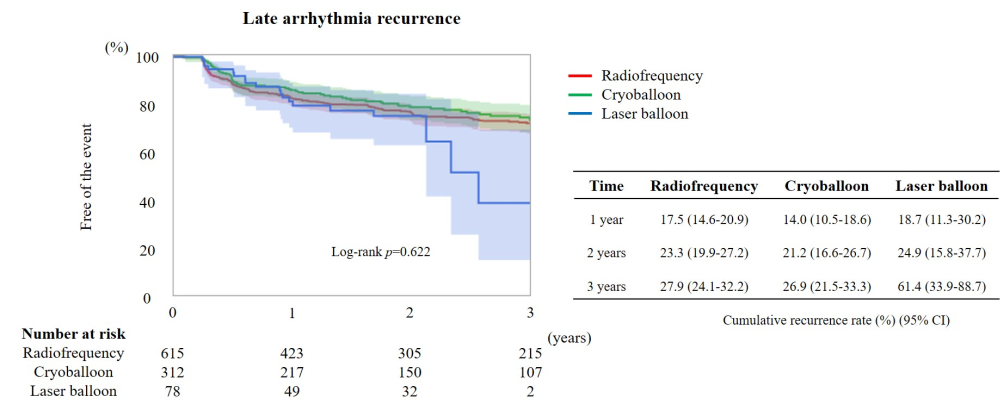

Supplement: Supplementary file 1 — Data S1: joa370285‐sup‐0001‐DataS1.zip. [file JOA3-42-e70285-s001.zip › supinfo/joa370285-sup-0003-FigureS2@Supplementary Figure 2.tif]
